# Involvement of MST1/mTORC1/STAT1 activity in the regulation of B‐cell receptor signalling by chemokine receptor 2

**DOI:** 10.1002/ctm2.887

**Published:** 2022-07-25

**Authors:** Yingzi Zhu, Heng Gu, Lu Yang, Na Li, Qiuyue Chen, Danqing Kang, Shengyan Lin, Yukai Jing, Panpan Jiang, Qianglin Chen, Li Luo, Ju Liu, Jiang Chang, Zhenzhen Li, Yi Wang, Xin Dai, Heather Miller, Lisa S. Westerberg, Chan‐Sik Park, Masato Kubo, Quan Gong, Lingli Dong, Chaohong Liu

**Affiliations:** ^1^ Department of Rheumatology and Immunology, Tongji Hospital, Tongji Medical College Huazhong University of Science and Technology Wuhan China; ^2^ Department of Pathogen Biology, Tongji Medical College Huazhong University of Science and Technology Wuhan China; ^3^ Department of Immunology, School of Medicine Yangtze University Jingzhou China; ^4^ Department of Research and Development BD Biosciences San Jose California United States; ^5^ Department of Microbiology Tumor and Cell Biology Karolinska Institutet Stockholm Sweden; ^6^ Department of Pathology, Asan Medical Center University of Ulsan College of Medicine Songpa‐gu Seoul Korea; ^7^ Laboratory for Cytokine Regulation, Center for Integrative Medical Science (IMS) RIKEN Yokohama Institute Kanagawa Japan

**Keywords:** autoimmune diseases, B‐cell receptor, CCR2, MST1, mTORC1

## Abstract

**Background:**

CCR2 is involved in maintaining immune homeostasis and regulating immune function. This study aims to elucidate the mechanism by which CCR2 regulates B‐cell signalling.

**Methods:**

In *Ccr2*‐knockout mice, the development and differentiation of B cells, BCR proximal signals, actin movement and B‐cell immune response were determined. Besides, the level of CCR2 in PBMC of SLE patients was analysed by bioinformatics.

**Results:**

CCR2 deficiency reduces the proportion and number of follicular B cells, upregulates BCR proximal signalling and enhances the oxidative phosphorylation of B cells. Meanwhile, increased actin filaments aggregation and its associated early‐activation events of B cells are also induced by CCR2 deficiency. The MST1/mTORC1/STAT1 axis in B cells is responsible for the regulation of actin remodelling, metabolic activities and transcriptional signalling, specific MST1, mTORC1 or STAT1 inhibitor can rescue the upregulated BCR signalling. Glomerular IgG deposition is obvious in CCR2‐deficient mice, accompanied by increased anti‐dsDNA IgG level. Additionally, the CCR2 expression in peripheral B cells of SLE patients is decreased than that of healthy controls.

**Conclusions:**

CCR2 can utilise MST1/mTORC1/STAT1 axis to regulate BCR signalling. The interaction between CCR2 and BCR may contribute to exploring the mechanism of autoimmune diseases.

## Graphical abstract

CCR2 deficiency upregulates mouse B‐cell proximal signalling, enhances MST1, mTORC1 and STAT1 signalling to promote metabolic activity and actin remodelling. CCR2 deficiency attenuates the T cell dependent immune response in mice. CCR2 deficiency may cause glomerular IgG deposition and autoantibodies in mice. The expression of CCR2 in peripheral blood B cells of SLE patients is decreased.

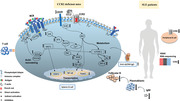

## 1 BACKGROUND

Exogenous antigens can induce B‐cell receptor (BCR) activation and signal cascade.^1^ BCR is coupled to disulphide heterodimers CD79a and CD79b non‐covalently, and then the proximal signalling molecules are activated.^2^ Syk can be recruited to phosphorylated immunoreceptor tyrosine‐based activation motifs, then activate BTK and PLCγ2.^3^ CD19 recruits BTK by activating PI3K, which is critical for amplifying downstream signalling. In different stages of BCR clustering, cytoskeleton actin can be targeted by B‐cell signalling to undergo characteristic remodelling, F‐actin accumulates near BCR microclusters in a polarised manner.^4^, ^5^, ^6^ In addition to antibody secretion, B cells perform multiple functions in the immune system, including antigen presentation and cytokine secretion.^7^

Mammalian target of rapamycin (mTOR) can facilitate B‐cell activation and autoantibody production,^8^ and the clinical efficacy of rapamycin in the treatment of rheumatism has been proved.^9^, ^10^ During B‐cell activation, MST1 functions as a molecular brake to balance immune tolerance, its depletion leads to hypergammaglobulinemia in mice.^11^ MST1‐deficient mice have decreased marginal zone (MZ) B cells and downregulated BCR clustering.^12^ Besides, clinical efficacy of JAK inhibitor supported the involvement of JAK/STAT pathway in the evolution of systemic lupus erythematosus (SLE).^13^

Chemokine (C‐C motif) receptor 2 (CCR2) interacts with its ligand CCL2 at different expression levels to induce the recruitment of chemotactic cells to tissues, maintain immune homeostasis and regulate autoimmunity.^14^, ^15^, ^16^ The decreased CCR2 expression in T cells is correlated to diseased activity of SLE,^17^ and CCR2 is involved in recruiting basophil to induce skin lesions in SLE patients.^18^ Functional and constitutive CCR2 can be expressed in B cells,^19^ and in the case of parasitic infection, B‐cell growth was enhanced in CCR2^–/–^ mice.^20^ CCR2 and CCL2 have been reported as negative homing regulators.^21^ However, the mechanism by which CCR2 regulates B‐cell signalling in immune disorders remains unclear.

Therefore, we used CCR2‐deficient mice to explore whether B‐cell signalling or related biologic activity will be affected by the absence of CCR2, and attempted to elucidate the molecular mechanisms involved.

## 2 METHODS AND MATERIALS

### 2.1 Animals

*Ccr2* knockout (KO) mice, wild type (WT) and μMT mice on C57BL/6J background were purchased from Jackson laboratory and bred in a SPF animal department. *Ccr2‐*KO mice were hybridised with WT mice to obtain same background heterozygous *Ccr2* littermates to be used as controls. Mice between 6‐ and 8‐week‐old were used. Animal experimentation was reviewed and approved by the Institutional Animal Care and Ethics Committee of Animal Experimentation of Tongji Medical College.

### 2.2 Blood samples from patients

Peripheral blood of 8 SLE patients from the outpatient department and 9 healthy controls (HCs) were collected. The experiments on human blood samples have been reviewed and approved by the Medical Ethics Committee of Tongji Hospital, Tongji Medical College, Huazhong University of Science and Technology. Ethical approval number: TJ‐IRB20210325. Signed informed consent were obtained.

### 2.3 Cell isolation and purification

The spleen of mouse was grinded and gradient centrifuged by Ficoll‐Hypaque solution for 20 min (room temperature, brake = 5) to obtain mononuclear cells. B cells were then purified from splenic mononuclear cells by incubation of anti‐Thy‐1 (105310, BioLegend) and guinea pig complement (C300‐0500, Rockland Immunochemicals) for 30 min (37°C), followed by incubation for 1 h to remove adherent cells, as described previously.^22^ Unilateral femoral and tibial bone marrow (BM) cavities were rinsed twice with 10 ml HBSS medium containing foetal bovine serum, after centrifugation, cells were lysed with Red Cell Lysis Buffer (RT122‐02, Tiagen) for 1–2 min, then washed and filtered. Ice‐cold PBS (5 ml) was injected into the mice peritoneal cavity and gently massaged for 1–2 min, fluid was collected and centrifuged to get cells.

For human blood sample, serum was removed after centrifugation at 3000 rpm for 10 min, the cell precipitate was suspended with PBS, and then slowly added into the tube of Ficoll‐Hypaque solution (ratio of 1:1). After centrifugation at 2000 rpm for 20 min (brake = 5), peripheral blood mononuclear cells (PBMC), granulocytes (PG), and white blood cells (PW) were obtained according to different densities.

### 2.4 Flow cytometry (FCM)

Following incubation with Fc blocker anti‐CD16/CD32 (101319, BioLegend), splenic mononuclear cells (2 × 10^6^), BM cells (2 × 10^6^) or peritoneal cavity cells (1 × 10^6^) were stained with antibodies (Abs) for 30 min. Abs were from BioLegend: FITC channel: anti‐B220 (103206), ‐CD127 (135008), ‐Annexin V (640906), ‐CD5 (100622), ‐CD19 (101506), ‐CD11b (101226). APC channel: anti‐CD43 (143208), ‐CD21 (123412). PE channel: anti‐BP‐1 (108307), ‐CD23 (101608). PE/Cy7 channel: anti‐CD24 (101822). PerCP/Cy5.5 channel: anti‐IgD (405710). Brilliant Violet (BV) 510 channel: anti‐B220 (103247), ‐CD45.2 (109838) and ‐CD138 (142521). BV421 channel: anti‐IgM (406518). AF647 channel: anti‐GL7 (144606). APC/Cy7 channel: anti‐CD45.1 (110716). From Biosearch Technologies: PE‐anti‐NP (N‐5070‐1).

For PBMC of SLE patients, after incubation with anti‐B220, cells were fixed and permeabilised, and then incubated with anti‐CCR2 (ab203128, Abcam).

For phosphoflow cytometry, after incubation with anti‐B220, cells were incubated with soluble antigen (sAg)‐10 μg/ml biotin‐conjugated F(ab')_2_ anti‐mouse Ig (M + G) (115‐066‐068, Jackson ImmunoResearch) at 4°C for 30 min. Twenty μg/ml streptavidin (16000114, Jackson ImmunoResearch) were added for 10 min and cells were activated at 37°C for 5, 10 and 30 min, respectively. Cells were fixed and permeabilised with Lyse/Fix buffer and Perm buffer III (558049, 558050, BD Biosciences), followed by incubation with anti‐pWASP (A300‐205A, Bethyl Laboratories) and AF488–phalloidin (R37110, Thermo Fisher), with or without AF405‐goat‐anti‐rabbit (G/R) IgG (A‐31556, Thermo Fisher).

Samples were analysed by Attune™ NxT sonic focused flow cytometer (Thermo Fisher) and FlowJo software (BD Biosciences).

### 2.5 Confocal microscopy (CFm) and total internal reflection fluorescence microscopy (TIRFm)

For CFm assay, purified splenic B cells (2.5 × 10^5^) were incubated with 10 μg/ml AF546 F(ab')_2_ anti‐mouse Ig (M + G) at 4°C for 30 min and activated at 37°C for 5, 10 and 30 min. For TIRFm assay, liposomes, streptavidin and biotinylated F(ab')_2_ were tethered and attached to the bottom of chambers to mimic membrane‐associated antigen (mAg), as described previously.^23^ Splenic B cells (5 × 10^5^) were stimulated with AF546‐mb Fab'‐goat‐anti‐mouse Ig (M + G) tethered to lipid bilayers at 37°C for 3, 5 and 7 min before staining with Abs.

Abs from Cell Signaling Technology: phosphorylated SHIP‐1 (pSHIP‐1) (3941S), pSTAT1 (9167S), pSTAT5 (4322S), and pNF‐κB (3033S). From Abcam: pBTK (ab52192), pCD19 (ab203615). From Thermo Fisher: F‐actin (R37110), AF488 G/R IgG (A‐11008), AF405 G/R IgG and AF405 G/M IgG (A‐31553). From Bethyl Laboratories: pWASP (A300‐205A). From Merck‐Millipore: protein phosphorylated tyrosine (pY) (05‐321).

Representative images were taken and analysed for mean fluorescence intensity (MFI), B‐cell contact area (under interference reflection microscopy, IRM), and colocalisation using NIS elements AR 5.01 software (Nikon). For CFm and TIRFm assay, 60× or 100× oil lens of microscopy were used respectively. Image resolution is 2048 × 2048. Data from three independent experiments using more than 30–50 individual cells were included for each parameter.

### 2.6 Immunoblotting

Purified splenic B cells (2 × 10^6^) were activated at 37°C with sAg for 5, 10 and 30 min. Cell lysates were obtained using a mixed RIPA buffer, as previously described.^24^ Lysates were run through SDS‐PAGE and western blotting. Abs from Cell Signaling Technology: BTK (8547S), pAKT (4060L), AKT (9272S), SHIP‐1 (2728S), pFOXO1 (9461S), FOXO1 (2880S), pS6 (4856S), S6 (2217S), pPI3K (4228S), PI3K (4292S), pMST1 (3681S), MST1 (PA5‐22015), pmTOR (5536S), mTOR (2983S), pEZRIN (3726S), STAT1 (14994S), P65 (4764S), pIKKB (2697S), IKKB (8943S), and anti‐human‐CCR2 (12199S). From Abcam: STAT5 (ab194898). From Santa Cruz Biotechnology: WASP (sc‐13139). Loading controls: anti‐mouse β‐actin (60008‐1‐IG‐10, Proteintech), anti‐human GAPDH (5174S, Cell Signaling Technology). Western blotting imaging was performed using the ChemiDoc™XRS + imaging systems (Bio‐Rad).

### 2.7 In vitro specific inhibition

Purified *Ccr2‐*KO splenic B cells (2 × 10^6^) were incubated with rapamycin (20 nM, HY‐10219, MedChem Express), XMU‐MP‐1 (3 μM, T4212, TargetMol), fludaradine (5 μg, T1038, TargetMol) or R406 (5 μM, T6174, TargetMol) at 37°C for 2 h (R406 for 4 h) before being activated with sAg. Then western blotting was performed on WT B, *Ccr2*‐KO B and *Ccr2*‐KO plus inhibitor B cells.

### 2.8 Seahorse analysis

Cell metabolism were detected by using Seahorse XFe24 cell metabolism analyser (XFe24, Agilent Technologies). For the measurement of oxygen consumption rate (OCR), purified splenic B cells (2 × 10^6^) were stimulated with F(ab')_2_ anti‐mouse Ig (M + G) for 2 h, then incubated with 1.5 μM oligomycin, 1 μM FCCP, 500 nM rotenone and 1 μM antimycin A sequentially in poly‐D‐lysine solution (50 μg/ml) (C0312, Beyotime) coated 24‐well plate. For the measurement of extracellular acidification rate (ECAR), after incubation with F(ab')_2_ Ig (M + G) overnight, cells were stimulated with 10 mM glucose, 2 μM oligomycin, 5 mM 2‐DG sequentially.

### 2.9 In vitro proliferation and apoptosis

Purified splenic B cells (2 × 10^6^) were incubated with 5 μM Celltrace Violet (CTV, C34557, Thermo Fisher) before being seeded into 96‐well plates with 250 μl complete RPMI1640 medium, as previously described.^24^ Lipopolysaccharide (LPS, 5 μg/ml) or C_P_G (10 μg/ml) were added into medium to stimulate cells for 72 h, F(ab')_2_ anti‐mouse Ig (M + G) (3 μg/ml), anti‐CD40 (10 μg/ml) and IL‐4 (5 ng/ml) were added to stimulate cells for 96 h. Cells apoptosis were measured after incubation with anti‐Annexin V and PI (70‐AP101‐100, Multiscience Biotech).

### 2.10 Calcium flux assay

Purified splenic B cells (5 × 10^6^) were labelled with 0.5 μM calcium‐sensitive dye Fluo‐4 AM (S1060, Beyotime) in Ca^2+^ free balanced salt solution for 25 min (37°C), and then stained with anti‐B220 Ab for 30 min. Using a LSRII flow cytometer (BD Biosciences), a baseline fluorescence intensity was recorded for the first 30 s, then cells were immediately stimulated with 10 μg/ml pre‐warmed F(ab')_2_ anti‐mouse Ig (M + G) for the next 270 s to analyse Ca^2+^ flux kinetics.

### 2.11 Scanning electron microscopy (SEM)

Incubate poly‐D‐lysine coated sterile coverslips with 10 μg/ml F(ab')_2_‐Ig (M + G) at 37°C for 3 h. Purified B cells (3 × 10^6^/ml) were added gently onto the coverslips for 10 min, then 400 μl 2.5% glutaraldehyde were added to terminate the stimulation and fix the cells for 20 min (on ice). Micrographs were captured using an Ultra‐high Resolution SEM (SU8010, HITACHI). Cellular filopodia expansion was imaged and the number and length of filopodia were quantified using ImageJ software (Bethesda).

### 2.12 Immunisation and enzyme‐linked immunosorbent assay (ELISA)

Mice were injected intraperitoneally (i.p.) with 40 μg 4‐hydroxy‐3‐nitrophenylacetyl conjugated keyhole limpet haemo‐cyanin (NP‐KLH) (N‐5060‐25, Biosearch Technologies), supplemented with Freund's incomplete adjuvant (S6322‐1VL, Sigma Aldrich). Fourteen days post‐prime, mice were boosted with the same reagents. Five days later, mice were euthanised and spleens were harvested. FCM was performed to test the proportion and cell number of B‐cell subsets. ELISA was performed using NP30‐bovine serum albumin (BSA)‐coated plates, according to the manufacturer's information on ELISA kit (Bethyl Laboratories) to detect the level of the NP‐specific IgM and IgG1 in immunised mice. Anti‐dsDNA IgG levels of unimmunised mice and chimeric mice were quantified by ELISA as previously described.^25^

### 2.13 Tissue immunofluorescence assay

Ten‐micrometre‐thick cryosections were obtained from frozen kidneys embedded in OCT medium (Tissue‐Tek, Sakura Finetek). Fix cryosections with ice‐cold acetone on slides for 5 min. Sections were incubated in 5% BSA (4240GR100, BioFroxx) with 1% anti‐CD16/CD32, and then incubated with AF488‐IgG (715‐545‐151, Jackson ImmunoResearch) overnight at 4°C. After rinse, images were captured by NIS elements AR 5.01 software (Nikon).

### 2.14 BM chimeras

BM cells from WT or *Ccr2*‐KO (CD45.2) mice and WT (CD45.1) mice were mixed at a 50:50 ratio. Recipient WT mice (CD45.1) were irradiated with 7 Gy X‐ray and injected with 5 × 10^6^ mixed cells through caudal vein under aseptic condition. One week before the radiation and 2 weeks after the injection, mice were given water with antibiotics (gentamicin and erythromycin). To verify the B‐cell‐specific effect of CCR2 deficiency, BM cells from WT or *Ccr2*‐KO mice and μMT mice were mixed at a 20:80 ratio, and then injected into irradiated recipient WT mice. FCM, immunohistochemistry and immunofluorescence were performed 8 weeks later.

### 2.15 RT‐PCR

According to the instructions of RNA Kit (AP‐MN‐MS‐RNA‐50, Axygen) and PrimeScript RT Reagent Kit (RR047A,Takara), total RNA of PBMC from SLE patients and HC was extracted and reverse transcribed. Then cDNA was assessed and the cycle threshold (ct) was used to quantify the transcription levels of *Ccr2*. Quantitative indicator was 2^−∆∆ct^. Primer sequences are: *ccr2* 5′ primer: agtcaactggaccaagccac and 3′ primer: tgtgaaaaaggcttctgaacttct

### 2.16 RNA‐sequencing

RNA‐sequencing was performed on the peripheral blood of SLE patients and HC. PBMC, PW and PG were sorted and washed in cold PBS and RNA was prepared. RNA libraries were constructed using Stranded mRNA sample preparation kit for Illumina. Fragmented mRNA pieces were copied into first‐strand cDNA, and then enriched to acquire the final cDNA library. Quality was examined using Agilent Tapestation 2200. The sequencing platform was Illumina Novaseq 6000, based on PE150 sequencing strategy, sequencing depth was 6G. The expression abundance of transcripts was standardised by fragments per kilobase of exon model per million mapped fragments (FPKM).

### 2.17 Statistical analysis

The normality of all the data was examined and two‐tailed unpaired Student's *t*‐test was used (Prism 8.0.1, GraphPad Software). Image J software was used for grey quantification of the immunoblotting stripes to compare protein expression levels. In FCM, the statistical analysis of BM subsets or peripheral B‐cell subsets was normalised according to the total number of cells in each corresponding tissue. Colocalisation was displayed using the Pearson's correlation coefficient. Data were extracted from at least three individual experiments, the figures shown are representative figures. Error bars were shown as mean (± SD). *p* < .05 represented significantly difference.

## 3 RESULTS

### 3.1 CCR2 deficiency disturbs the peripheral differentiation of B cells

**FIGURE 1:**
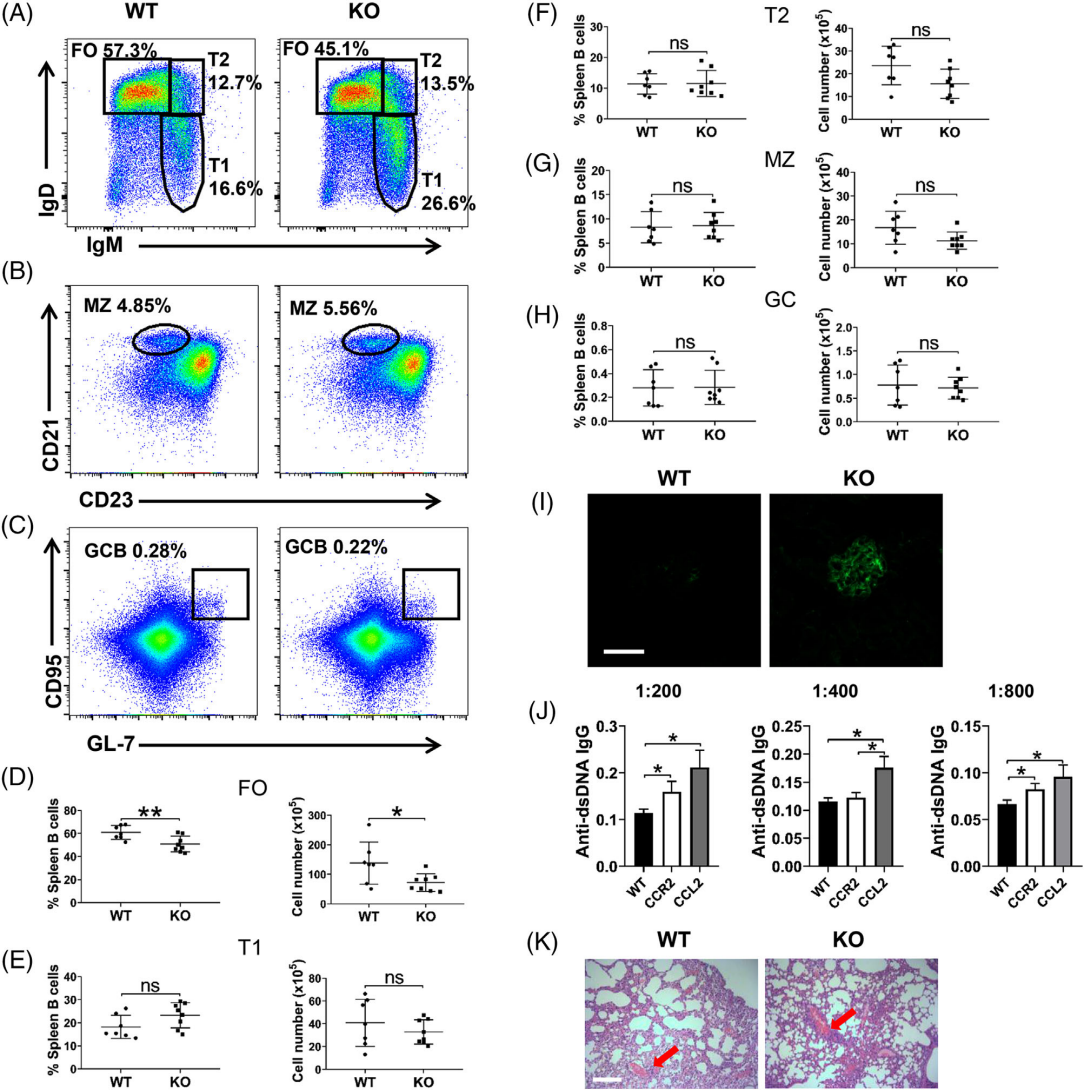
Peripheral differentiation of B cells is disturbed in *Ccr2*‐KO mice. (A–C) Representative dot plots of peripheral B‐cell subsets including follicular (FO) (B220^+^IgM^high^IgD^high^), transitional type‐1 (T1) (B220^+^IgM^high^IgD^low^), transitional type‐2 (T2) (B220^+^IgM^high^IgD^high^), marginal zone (MZ) (B220^+^CD23^low^CD21^high^), and germinal centre (GC) (B220^+^GL‐7^high^CD95^high^) B cells. Shown in the box is the proportion of each B‐cell subset. (D–H) Quantitative analysis of percentage and cell number of peripheral B subsets (WT *n =* 7, KO *n =* 8). (I) Representative immunofluorescent analysis (60 × objective, scale bar = 50 μm) of IgG deposits in glomeruli. (J) Serum anti‐dsDNA IgG levels of WT mice, *Ccr2*‐KO mice and *Ccl*2‐KO mice with serum dilution of 1:200, 1:400 and 1:800 (*n =* 9). (K) Haematoxylin and eosin (HE) staining of lung anatomical structure (10× objective, scale bar = 200 μm), the red arrows indicated lymphocytic infiltration around blood vessels. Error bars were shown as mean (± SD). Each symbol represents a mouse. *p < .05, **p < .01, ns: no statistical significance

The expression level of CCR2 on BM subsets was as follows: early‐pre‐B cell (c) > pro‐B cell (b) > pre‐pro‐B cell (a) > immature B cell (e) ≈ late‐pre‐B cell (d) >recirculating mature B cell (f), and the expression level of CCR2 on splenic B subsets was as follows: MZ > transitional type‐1 (T1) ≈ transitional type‐2 (T2) > follicular (FO) B cell (Figure S1A and B). CCR2 expression on B1 cells was significantly higher than that on peritoneal cavity B2 cells, no difference in CCR2 expression between B1a and B1b cells was found (Figure S1C). In BM, CCR2 deficiency had little impact on B‐cell development in *Ccr2*‐KO mice (Figure S1D–F). However, by detecting splenic B‐cell subsets (Figure 1A–C), we found that *Ccr2*‐KO mice had lower proportion and number of FO B cells than WT mice (Figure 1D). For T1, T2, MZ and germinal centre (GC) B cells, no significant difference between WT and *Ccr2*‐KO mice was found (Figure 1E‐H). Besides, there was no difference in peritoneal cavity B2, B1a and B1b cells (Figure S1G–L). To eliminate the potential effect of other immune cells, especially T cells, on the abnormal differentiation in *Ccr2*‐KO mice, irradiated CD45.1 recipient mice were injected with mixed BM cells from WT or KO mice and CD45.1 mice, a decrease of FO B cells in the CD45.2 population verified the inherent decrease of FO B cells in CCR2‐deficient mice (Figure S2A–E). However, we also found a decrease of FO B cells in CD45.1 population (Figure S2F and G). The significant IgG deposition in the glomeruli of *Ccr2*‐KO mice accompanied by increased level of anti‐dsDNA IgG (Figure 1I and J) was observed, and there was no difference between male and female mice (Figure S3A). Besides, *Ccr2*‐KO mice showed more severe lymphocyte infiltration in the lungs (Figure 1K), but not in liver, kidney and colon (Figure S3B). Further, irradiated WT mice were injected with mixed BM cells from WT or *Ccr2*‐KO mice and μMT mice. The decreased proportion and cell number of FO B cells and the decreased number of other peripheral B‐cell subsets confirmed the B‐cell‐specific effect of CCR2 on mice (Figure S4A–N). Similarly, glomerular IgG deposition and lung lymphocytic infiltration were more severe in *Ccr2‐*KO chimeras than WT chimeras (Figure S4O–Q). Collectively, CCR2 plays a crucial part in regulating B‐cell peripheral differentiation and maintaining the integrity of peripheral autoimmunity.

### 3.2 BCR proximal signalling is upregulated in *Ccr2*‐KO mice

**FIGURE 2:**
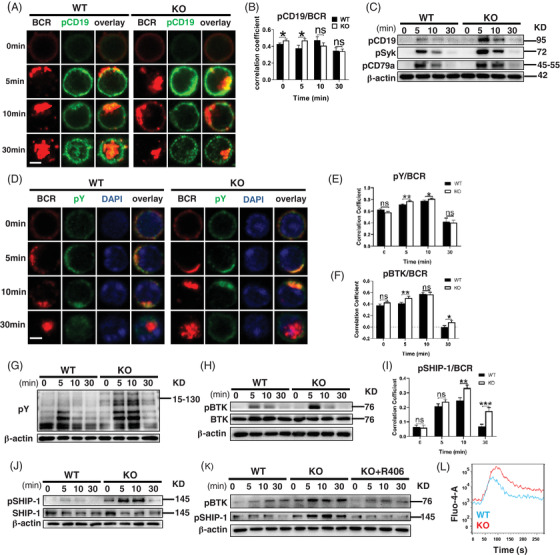
*Ccr2*‐KO mice exhibit enhanced BCR proximal signalling. Purified splenic B cells were incubated with AF546‐F(ab')_2_‐anti‐mouse‐Ig (M + G) at 4°C for 30 min and activated at 37°C for 5, 10 and 30 min, confocal microscopy (CFm) was performed. Cells were incubated with biotin‐conjugated F(ab')_2_‐anti‐mouse‐Ig (M + G) and streptavidin, then activated at 37°C for 5, 10 and 30 min, western blotting was performed. (A) Representative CFm images of phosphorylated CD19 (pCD19) and BCR (60× objective, scale bar = 2.5 μm). (B) Colocalisation between pCD19 and BCR. (C) Western blotting of pCD19, pSyk, pCD79a expression in B cells. (D) Representative CFm images of pY and BCR (60× objective, scale bar = 2.5 μm). (E) Colocalisation between pY and BCR. (F) Colocalisation between pBTK and BCR. (G) Western blotting of pY expression in B cells. (H) Western blotting of pBTK and BTK expression in B cells. (I) Colocalisation between pSHIP‐1 and BCR. (J) Western blotting of pSHIP‐1 and SHIP‐1 expression in B cells. (K) Western blotting of pBTK and pSHIP‐1 in WT B cells, *Ccr2*‐KO B cells and *Ccr2*‐KO B cells treated with 5μM R406. (L) Representative image of intracellular Ca^2+^ flux kinetics in WT and *Ccr2*‐KO B cells following stimulation with 10 μg/ml biotin‐conjugated F(ab')_2_ anti‐mouse Ig (M + G). All images were representative images from three independent experiments. The number of cells analysed for each parameter in CFm assay was 30–50. Error bars were shown as mean (± SD). *p < .05, **p < .01, ***p < .001, ns: no statistical significance

Upon stimulation with sAg, the colocalisation between BCR and pCD19 in *Ccr2*‐KO B cells was significantly increased at 5 min and gradually decreased to the 30 min time‐point when BCR is internalised (Figures 2A and B and S5A). Notably, although the colocalisation between pCD19 and BCR after 10 min of stimulation decreased in *Ccr2*‐KO B cells, the aggregation of pCD19 remained at higher levels than that in WT B cells. This indicates that CCR2 deficiency may affect not only the temporal but also the spatial organisation of CD19. The levels of pCD19, pCD79a and pSyk in B cells were increased under CCR2 deficiency (Figures 2C and S5B–D). Next, we evaluated the total BCR signaling level‐pY, the level of phosphorylated BTK and BCR signaling negative regulator SHIP‐1. Compared to WT B cells, the colocalisation between pY and BCR in CCR2‐deficient B cells was increased from 5 to 10 min after stimulation (Figure 2D and E), and that between pBTK and BCR was increased at 5 min after stimulation (Figures 2F and S5E). The expression of pY (Figures 2G and S5F and G) and pBTK (Figures 2H and S5H and I) in *Ccr2*‐KO B cells was increased. Similarly, the level of pSHIP‐1 in *Ccr2*‐KO B cells also increased after 5 and 10 min of activation (Figure S5J and K), and its colocalisation with BCR peaked at 10 min (Figure 2I). A higher expression of pSHIP‐1 in *Ccr2*‐KO B cells was also verified by immunoblotting (Figures 2J and S5L). Syk inhibitor R406 was used to block BCR signal, and the elevated pBTK and pSHIP‐1 caused by CCR2 deficiency were rescued (Figure 2K).

PLCγ2‐induced/calcium (Ca^2+^) mobilisation is responsible for mediating downstream signalling after BCR activation, PLCγ2/Ca^2+^ activity can modulate subsequent intranuclear signalling.^26^ Thus, enhanced Ca^2+^ mobilisation in *Ccr2‐*KO B cells upon F(ab')_2_ stimulation was observed (Figure 2L).

To sum up, BCR proximal signalling molecules were upregulated in the absence of CCR2, accompanied by enhanced intracellular Ca^2+^ activity after BCR activation.

### 3.3 Energy metabolism mediated by PI3K/AKT/mTORC1 is enhanced in CCR2‐deficient B cells

**FIGURE 3:**
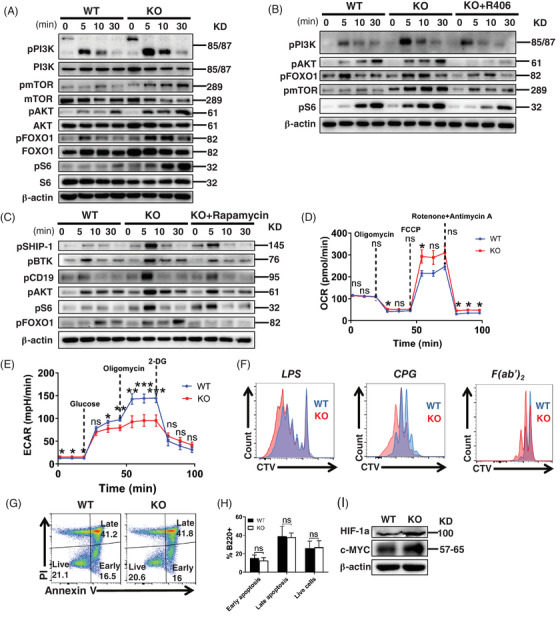
CCR2 deficiency enhances B‐cell metabolic process through the regulation on PI3K/AKT/mTORC1 signalling pathway.(A) Western blotting of pPI3K, PI3K, pAKT, AKT, pFOXO1, FOXO1, pS6, S6, pmTOR and mTOR levels in splenic B cells activated with biotin‐conjugated F(ab')_2_‐anti‐mouse‐Ig (M + G) and streptavidin. (B) Western blotting of pPI3K, pAKT, pFOXO1, pmTOR and pS6 in WT B cells, *Ccr2*‐KO B cells and *Ccr2*‐KO B cells treated with 5 μM R406. (C) Western blotting of pSHIP‐1, pBTK, pCD19, pAKT, pFOXO1 and pS6 levels in WT B cells, *Ccr2*‐KO B cells and *Ccr2*‐KO B cells treated with 20 nM rapamycin. (D) OCR detection of WT and *Ccr2*‐KO B cells. (E) ECAR detection of WT and *Ccr2*‐KO B cells. (F) Celltrace Violet (CTV) dilution of in vitro LPS‐stimulated (5 μg/ml), C_P_G (10 μg/ml)‐stimulated and F(ab')_2_ anti‐mouse Ig (M + G) (3 μg/ml) plus anti‐CD40 (10 μg/ml)‐stimulated B‐cell proliferation. (G) Representative dot plots of in vitro apoptosis of B cells following 96 h stimulation with F(ab')_2_ anti‐mouse Ig (M + G) (3 μg/ml) plus anti‐CD40 (10 μg/ml). (H) Quantification of percentage of apoptotic cells. (I) Western blotting of HIF‐1a and c‐MYC levels in WT and *Ccr2*‐KO B cells. All images were representative images from three independent experiments. Error bars were shown as mean (± SD). *p < .05, **p < .01, ***p < .001, ns: no statistical significance

One of the subsequent effects of BCR activation is metabolic activity. Cell differentiation‐associated molecule mTOR complex 1 (mTORC1) can mediate HIF‐1 expression to consequently influence the cell energy metabolism process via the PI3K/AKT pathway.^27^ Following sAg stimulation and specific Abs incubation, the expression of phosphorylated PI3K (pPI3K), pAKT, pS6, pmTOR and AKT distal glucose metabolism‐related transcriptional factor (pFOXO1) was increased in CCR2‐deficient B cells (Figures 3A and S6A–E). Second, similar to the level of BCR signal blocked by Syk inhibitor R406 (Figure 3B), the expression of pPI3K, pAKT, pFOXO1, pS6 and the proximal signalling molecules pSHIP‐1, pBTK and pCD19 in CCR2‐deficient B cells incubated with rapamycin, an mTORC1 inhibitor, could be rescued to the level of WT mice (Figure 3C). This indicates that mTORC1 is necessary for CCR2‐regulated B‐cell metabolism. In addition, following stimulation with F(ab')_2_‐Ig (M + G), seahorse assay showed that *Ccr2‐*KO B cells had higher ATP production levels and maximal respiratory capacity than WT B cells (Figure 3D), however, the glycolysis ability of *Ccr2‐*KO B cells was decreased (Figure 3E). Besides, upon in vitro LPS, C_P_G or F(ab')_2_‐Ig (M + G) stimulation, *Ccr2*‐KO B cells showed a faster proliferation (Figure 3F), although no significant change in the apoptosis was found (Figure 3G and H).

BCR induces heightened aerobic glycolysis by promoting glucose and oxygen utilisation, and B‐cell activation drives Myc‐dependent upregulation of glucose transporter 1 and HIF‐1α‐mediated upregulation of oxygen transport.^28^ In immunoblotting analysis, *Ccr2*‐KO B cells exhibited increased levels of HIF‐1α and c‐MYC (Figure 3I).

Taken together, in the metabolic activity after BCR activation, CCR2 mainly interacts with PI3K/AKT/mTORC1 pathway to regulate the B cells metabolic signal.

### 3.4 CCR2 deficiency promotes MST1‐regulated F‐actin accumulation and BCR internalisation

**FIGURE 4:**
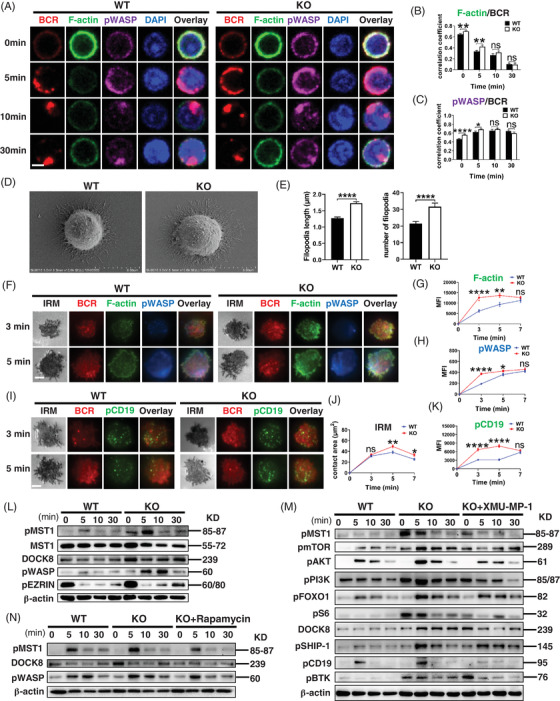
*Ccr2‐*KO mice exhibit increased accumulation of F‐actin through the enhancement of the MST1/mTORC1/DOCK8/WASP pathway. (A) Representative CFm images of pWASP, F‐actin and BCR (60× objective, scale bar = 2.5 μm). (B) Colocalisation between F‐actin and BCR. (C) Colocalisation between pWASP and BCR. (D) Filopodia dilatation visualised using electron microscopy (scale bar = 5 μm). (E) Quantitative analysis of number and length of the filopodia. (F) Representative total internal reflection fluorescence microscopy (TIRFm) images of F‐actin and pWASP at 3 and 5 min of activation (100× objective, scale bar = 2.5 μm). (G) The MFI of F‐actin. (H) The MFI of pWASP. (I) Representative TIRFm images of pCD19 at 3 and 5 min of activation (100× objective, scale bar = 2.5 μm). (J) Quantitative analysis of B‐cell area in the contact zone (under interference reflection microscopy). (K) Quantitative analysis of pCD19 MFI. (L) Western blotting of DOCK8, pMST1, MST1, pWASP and pEZRIN levels. (M) Western blotting of DOCK8, pMST1, pSHIP‐1, pBTK, pCD19, pmTOR, pAKT, pPI3k, pFOXO1 and pS6 protein expression levels in WT, *Ccr2*‐KO B cells and *Ccr2*‐KO B cells treated with XMU‐MP‐1. (N) Western blotting of DOCK8, pMST1 and pWASP levels in WT, *Ccr2*‐KO B cells and *Ccr2*‐KO B cells treated with rapamycin. All images were representative images from three independent experiments. Error bars were shown as mean (± SD). *p < .05, **p < .01, ****p < .0001, ****p < .0001, ns: no statistical significance

During sAg‐induced BCR clustering, cortex actin is detached from the membrane, and F‐actin accumulates near BCR microclusters in a polarised manner.^5^, ^6^ The F‐actin levels observed in activated CCR2‐deficient B cells were consistently higher than that in WT B cells (Figure 4A and S7A), as well as the colocalisation between F‐actin and BCR after 5 min of stimulation (Figure 4B). Similarly, the level of pWASP, the actin nucleation‐promoting factor and the colocalisation between pWASP and BCR in sAg stimulated *Ccr2*‐KO B cells were also increased (Figure 4C and S7B). Phosphoflow cytometry demonstrated the expression of F‐actin and pWASP increased at 10 and 5 min under sAg stimulation, respectively (Figure S7C and D). Additionally, more detailed morphologic changes of B cells were obtained by SEM (Figure 4D). The number and length of filopodia were remarkably elevated in *Ccr2*‐KO B cells upon sAg stimulation (Figure 4E).

Compared with CFm, TIRFm has a higher resolution when taking spatial pictures of B cells at the moment of contact with mAg. The level of F‐actin in the contact zone of KO B cells increased compared with WT B cells after 3–5 min of mAg stimulation (Figure 4F and G). Consistent with the augmented F‐actin accumulation, the level of pWASP in CCR2‐deficient B cells was also elevated at 3 min after stimulation (Figure 4H). Moreover, these changes were accompanied by the increased accumulation of pCD19 in CCR2‐deficient B cells at 3 and 5 min of stimulation (Figure 4I–K), and pY, pBTK and pSHIP‐1 were highly recruited in CCR2‐deficient B cells (Figure S7E–I).

MST1 regulates cytoskeletal microtubule dynamics and promotes F‐actin polymerisation^29^ and also modulates BCR to induce actin remodelling by attaching WASP.^30^ In the absence of CCR2, the levels of pMST1, pWASP and DOCK8 were increased after sAg stimulation (Figures 4L and S7J–M). After pre‐treatment with MST1 inhibitor, XMU‐MP‐1, the increased expression of pmTOR, pAKT, pS6 and DOCK8 in *Ccr2*‐KO B cells were rescued (Figure 4M). And also, pre‐treatment with rapamycin can rescue the elevation of DOCK8 and pWASP levels in CCR2‐deficient B cells (Figure 4N), suggesting the suppression of mTORC1 in CCR2‐deficient B cells can influence the DOCK8/WASP axis. Taken together, CCR2 deletion promoted actin accumulation and BCR internalisation through the activity of MST1/mTORC1/DOCK8/WASP axis.

### 3.5 CCR2 depletion triggers the activation of STAT1 to enhance BCR signalling

**FIGURE 5:**
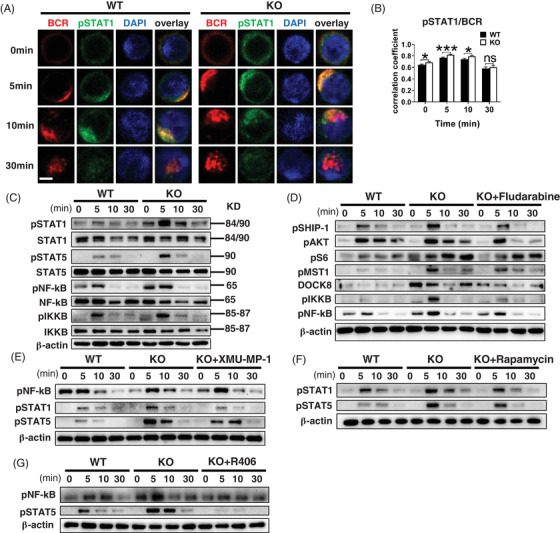
CCR2 deficiency triggers the activation of STAT1 to enhance BCR signalling. (A) Representative CFm images of pSTAT1 (60× objective, scale bar = 2.5 μm). (B) Colocalisation between pSTAT1 and BCR. (C) Western blotting of pSTAT1, STAT1, pSTAT5, STAT5, pNF‐κB, NF‐κB, pIKKB and IKKB in WT and *Ccr2*‐KO splenic B cells. (D) Western blotting of pSHIP‐1, pAKT, pS6, pMST1, DOCK8, pIKKB and pNF‐κB in WT, *Ccr2*‐KO B cells and *Ccr2*‐KO B cells treated with fludarabine. (E) Western blotting of pNF‐κB, pSTAT1 and pSTAT5 in WT, *Ccr2*‐KO B cells and *Ccr2*‐KO B cells treated with XMU‐MP‐1. (F) Western blotting of pSTAT1 and pSTAT5 in WT, *Ccr2*‐KO B cells and *Ccr2*‐KO B cells treated with rapamycin. (G) Western blotting of pNF‐κB and pSTAT5 in WT B cells, *Ccr2*‐KO B cells and *Ccr2*‐KO B cells treated with 5 μM R406. All images were representative images from three independent experiments. Error bars were shown as mean (± SD). *p < .05, ***p < .001, ns: no statistical significance

During BCR signal transduction, intracellular signal transduction can be connected with intranuclear signal regulation through activation of transcription factors.^31^ CCR2 is associated with the activity of the JAK/STAT pathway and its downstream transcriptional factors,^32^ and the inhibition on the CCL2/CCR2 axis can affect the activity of NF‐κB.^33^ Upon sAg stimulation, the interaction between pSTAT1 and BCR in CCR2‐deficient B cells significantly increased within 10 min after stimulation (Figures 5A and B and S8A). And the interaction between pNF‐κB or pSTAT5 and BCR was also higher in CCR2‐deficient B cells at 5 min (Figure S8B–G). Increased expression of pSTAT1, pSTAT5 and pNF‐κB in CCR2‐deficient B cells was also found at the protein level (Figures 5C and S8H–K).

Previous study showed that MST1 regulates the AKT/mTOR pathway^34^ and mediates the activation of JAK/STAT downstream transcription factors.^35^ After pre‐treated with STAT1 inhibitor, fludarabine, the levels of pAKT, pS6, DOCK8, pNF‐κB, and pIKKB in *Ccr2*‐KO B cells were rescued (Figure 5D). Further, following pre‐treatment with XMU‐MP‐1 or rapamycin, the upregulated expression of pSTAT1 and pSTAT5 in CCR2‐deficient B cells was rescued to the similar level in WT B cells (Figure 5E and F). Similarly, elevated levels of pNF‐κB and pSTAT5 in *Ccr2*‐KO B cells can be rescued by R406 (Figure 5G). Mechanistically, according to the variation trend of molecule signal after the inhibition of MST1, mTORC1 and STAT1 pathways, we suggested that in CCR2‐deficient mice, a sequential activity of MST1/mTORC1/STAT1 is involved in the regulation on BCR signalling.

### 3.6 CCR2 deficiency attenuates the response of mice to T‐cell‐dependent antigens

**FIGURE 6:**
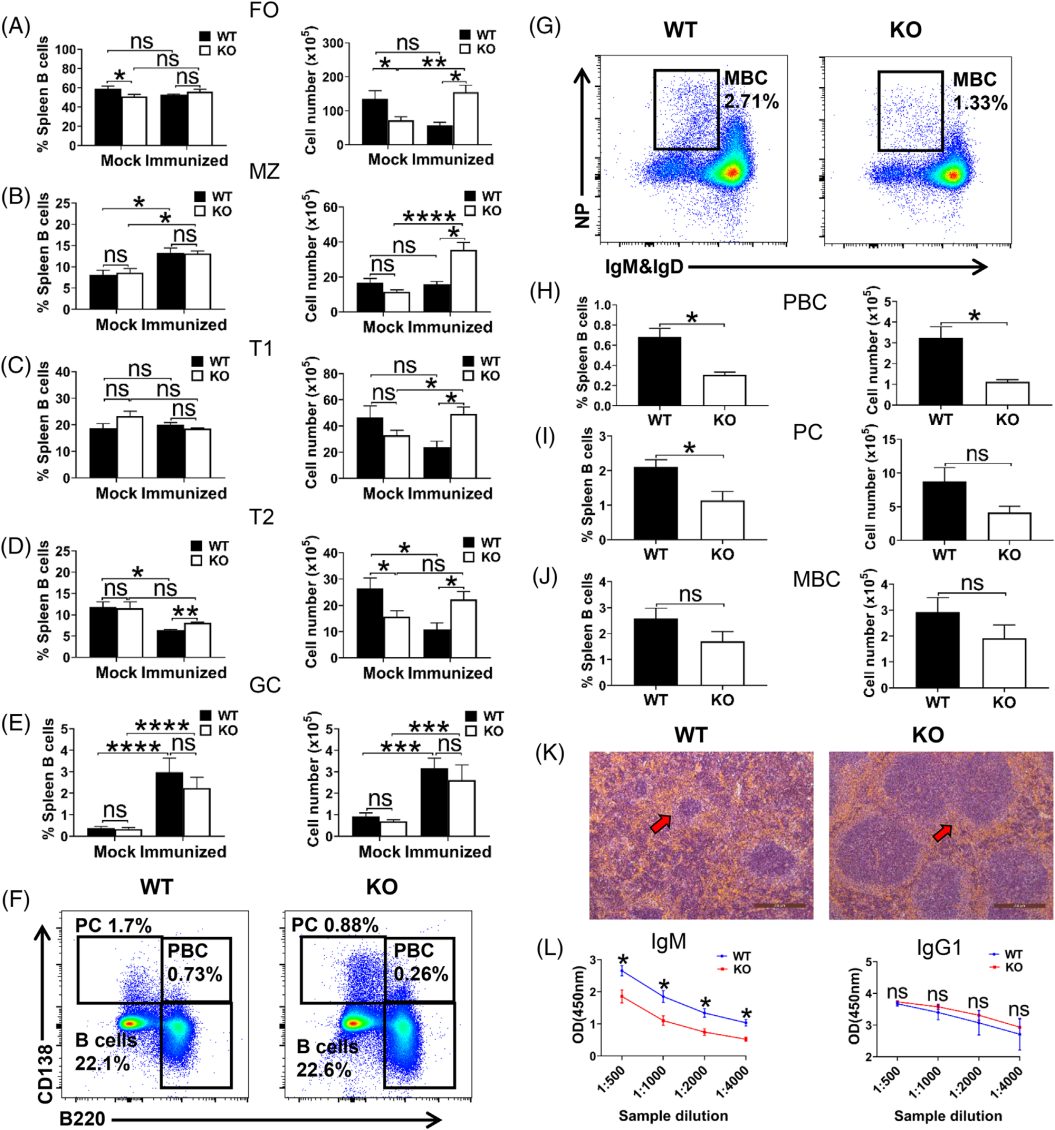
*Ccr2*‐KO mice generate fewer plasma cells and produce less antigen‐specific antibodies during T‐cell‐dependent immune response. WT and *Ccr2*‐KO mice were injected intraperitoneally (i.p.) with T‐cell‐dependent antigen 4‐hydroxy‐3‐nitrophenylacetyl conjugated keyhole limpet haemo‐cyanin (NP‐KLH, 40 μg/mouse). Mice were boosted with the same reagents 14 days later. (A–E) Quantitative analysis of the proportion and cell number of FO, MZ, T1, T2 and GC B cells. (F and G) Representative dot plots of plasmablasts (PBC), plasma cells (PC) and memory B cells (MBC). (H–J) Quantitative analysis of the proportion and cell number of PBC, PC and MBC. (K) Images of HE stained spleens from immunised mice (10× objective, scale bar = 200 μm). (L) ELISA was performed on serum extracted from immunised WT and *Ccr2‐*KO mice, NP‐specific IgM and IgG1 levels were quantified. Error bars were shown as mean (±SD). *p < .05, **p < .01, ***p < .001, ****p < .0001, ns: no statistical significance

Based on the upregulation of BCR‐related signalling molecules by CCR2 deletion, the impact of CCR2 on B‐cell function were explored by immunising WT and *Ccr2*‐KO mice with T‐cell‐dependent antigen. After immunisation with 40 μg NP‐KLH, spleens of mice were harvested to determine the peripheral B‐cell subsets and antibody‐secreting cells. In contrast to unimmunised condition, immunised *Ccr2*‐KO mice exhibited increased number of FO B, MZ B, T1 and T2 cells than that in WT mice (Figure 6A–D). There was no alteration in GC B cells before and after immunisation (Figure 6E). Moreover, the proportion and/or number of plasmablasts (PBC) and plasma cells (PC) were reduced in *Ccr2*‐KO mice (Figure 6F–I). We also noticed a downward trend in the proportion and number of memory B cells (MBC) (Figure 6J).

Besides, enlarged lymphatic follicles structures in the spleens of immunised *Ccr2*‐KO mice were observed (Figure 6K), and NP‐specific IgM level in CCR2‐deficient mice was significantly reduced (Figure 6L). In summary, the absence of CCR2 was sufficient to induce immune dysfunction in mice.

### 3.7 CCR2 expression is decreased in PBMC of SLE patients

**FIGURE 7:**
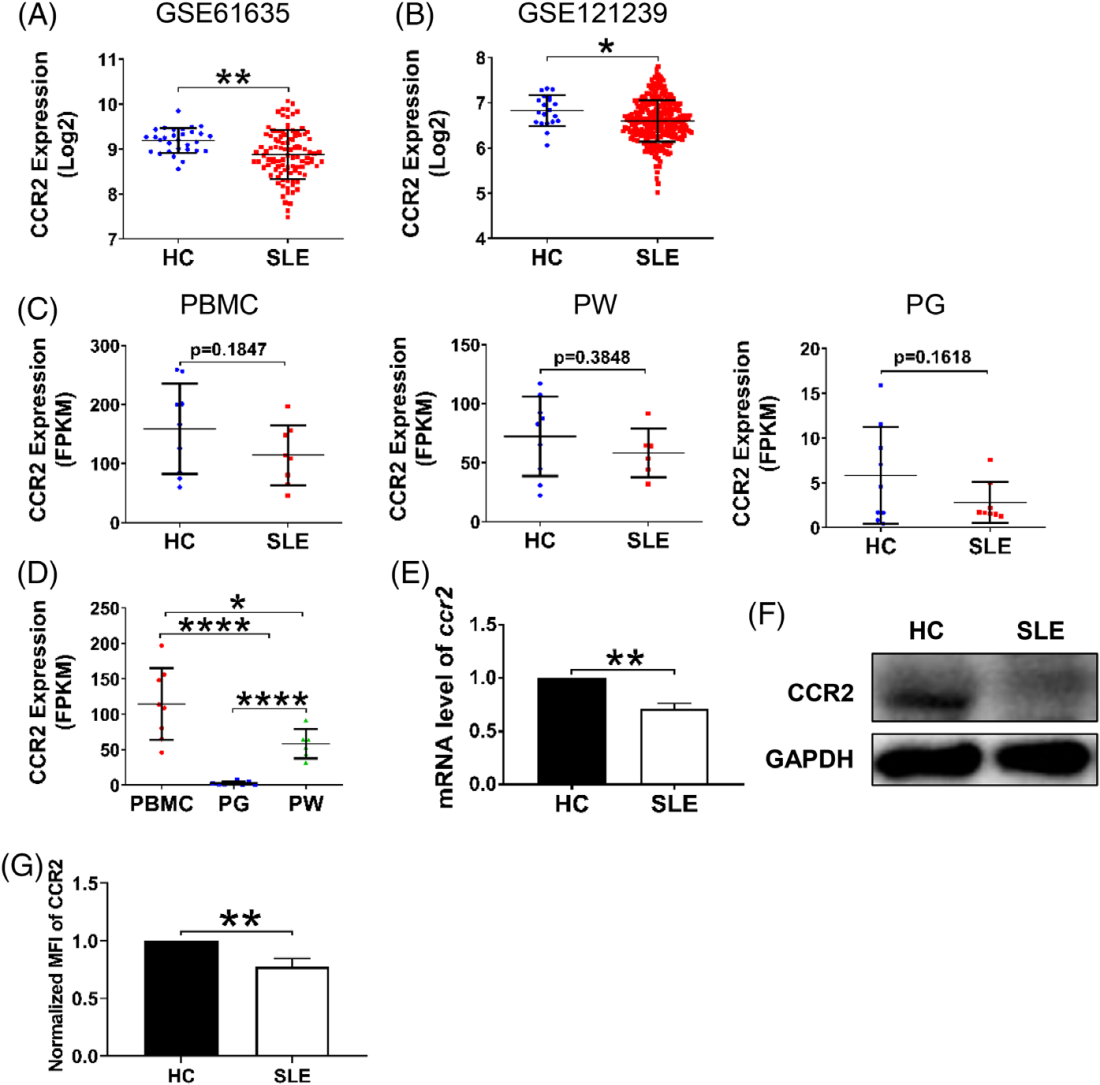
CCR2 expression is decreased in peripheral blood B cells of SLE patients. By using Gene Expression Omnibus (GEO) database platform of National Center for Biotechnology Information (NCBI), we analysed the expression level of CCR2 in the peripheral blood of SLE patients. (A) GSE61635 set was screened from GEO database. Expression of CCR2 in the whole peripheral blood of SLE patients (*n =* 99) and healthy controls (HC) (*n =* 30). (B) GSE121239 set was screened from GEO database. Expression of CCR2 in peripheral blood mononuclear cells (PBMC) of SLE patients (*n =* 292) and HC (*n =* 20). (C) RNA‐sequencing on expression of CCR2 in PBMC (*n =* 8), peripheral white blood cells (PW) (*n =* 6) and peripheral blood granulocytes (PG) (*n =* 8) of SLE patients and HC (*n =* 9). Blood samples were collected from outpatients. The expression of CCR2 is quantified as fragments per kilobase of exon model per million mapped fragments (FPKM). (D) Comparison of CCR2 expression levels in PBMC, PW and PG of SLE patients. (E) The normalised mRNA level of *ccr2* was detected by real‐time PCR. (F) Western blotting of CCR2 expression in PBMC of SLE patients and HC. (G) The expression of CCR2 in the peripheral blood B cells of SLE patients detected by flow cytometry. Error bars were shown as mean (± SD). Each symbol represents one patient. *p < .05, **p < .01, ****p < .0001

Lastly, we investigated the level of CCR2 in the peripheral blood of SLE patients. First, by using GEO database platform of NCBI, we analysed the level of CCR2 in the peripheral blood of SLE patients and HCs in currently available microarray data sets of relatively large sample size. For the whole peripheral blood, data from GSE61635 set (SLE *n =* 99, HC *n =* 30)^36^ showed that the level of CCR2 in the peripheral blood of SLE patients was lower than that of HCs (*p* = .0029) (Figure 7A). For PBMC, data from GSE121239 set (SLE *n =* 292, HC *n =* 20)^37^, ^38^ also showed a decreased level of CCR2 in PBMC of SLE patients, compare to HCs (*p* = .0323) (Figure 7B). Then, we performed bioinformatics analysis on the expression of CCR2 in the collected outpatient samples. RNA sequencing showed that there was a downward trend in terms of the expression of CCR2 in PBMC, PW and PG of SLE patients (Figure 7C), compared to HCs, although not statistically significant. In addition, CCR2 expression also varied among the three types of cells, with the highest expression of CCR2 in PBMC (Figure 7D). Meanwhile, we detected the level of *ccr2* by RT‐PCR, results verified a decreased expression of *ccr2* in PBMC of SLE patients (Figure 7E). Also, in PBMC or peripheral blood B cells of SLE patients, western blotting and FCM showed a decrease of CCR2 expression (Figure 7F and G). Thus, CCR2 expression was reduced at multiple levels in SLE patients.

## 4 DISCUSSION

So far, the interaction between CCR2 and BCR signalling has been poorly studied. In this study, we innovatively illustrated the phenotype and biological changes of B cells in CCR2‐deficient mice. CCR2 deficiency upregulates key BCR proximal signalling, increases MST1 level acting on mTORC1 and STAT1, thereby promoting actin remodelling, B‐cell metabolic proliferation and B cells downstream transcriptional signalling. B‐cell immune response in CCR2‐deficient mice appeared to be weakened and IgM levels decreased. In addition, the expression of CCR2 on the peripheral blood B cells of SLE patients was lower than that of healthy patients.

CCR2 is involved in both immune homeostasis and immune dysregulation. The entry of monocytes from BM into the peripheral circulation requires the chemotactic function of CCR2.^39^ SLE susceptibility gene *IRF5* can regulate the expression of CCR2 in Ly6C monocytes, thus affecting the migration of monocytes to the peritoneal cavity.^40^ In addition, CCR2 deficiency affects not only monocytes or macrophages and T‐cell infiltration in lupus kidney but also systemic T‐cell response in lupus mice.^41^ CCR2 expression is significantly elevated in plasma cells of bronchoalveolar lavage fluid of patients infected with SARS‐CoV‐2.^42^ MHC‐II expression on CCR2 negative myocardial macrophages can be regulated by B cells,^43^ and inhibition of B cells in CCR2‐deficient arthritic mice alleviates arthritic symptoms.^44^

B‐cell proliferation plays an important role in the pathogenesis of SLE, B‐cell components are severely disrupted in lupus patients.^45^ Accelerated apoptosis and dysfunction of atypical memory B cells have been reported in SLE patients.^46^ Cyclophosphamide can treat SLE by targeting activated B cells that are undergoing proliferation,^47^ monoclonal antibodies to CD20 and CD22 have been used to deplete B cells in SLE patients and lupus mice.^48^ In this study, the reduced expression of CCR2 in peripheral B cells of SLE patients was noticed, and the metabolic activity of B cells in CCR2‐deficient mice was significantly increased, thus promoting the development of autoimmunity. This was also reflected by the significant deposition of IgG immune complex in the glomeruli and increased anti‐dsDNA IgG level in CCR2‐deficient mice.

MTORC1 activation has become an important pathologic pathway in SLE and other autoimmune diseases. Blocking mTORC1 pathway can inhibit T‐bet expressing B‐cell generation,^46^ lymphocyte activation and progression of lupus nephritis of SLE prone mice.^49^ Inhibition of the mTOR pathway also reduces the production of anti‐dsDNA antibodies produced by B‐cell immune disorders.^50^ In addition, inhibition of the JAK2/STAT1 pathway in lupus nephritis mice can alleviate renal function damage and immune complex deposition and reduce the level of proteinuria and anti‐dsDNA IgG.^51^ In this study, both mTORC1 and STAT1 signalling were enhanced in CCR2‐deficient mice, promoting B‐cell metabolism and transcriptional signalling, thus suggesting the role of this mechanism in autoimmunity.

CCL2 has been confirmed as a negative regulator of BCR signal.^24^ In this study, we complemented the role of CCR2, thus linking CCL2‐CCR2 and clarifying the role of this axis in BCR signalling. Defects in the CCL2‐CCR2 signalling axis upregulate B‐cell signalling, actin remodelling and B‐cell metabolic activity in mice. BCR signal strength can affect the tendency of B cells to differentiate into FO or MZ groups.^52^ CCL2 deficiency leads to decreased MZ B cells and increased spontaneous germinal centres, while CCR2 deficiency leads to fewer FO B cells. In adaptive immune response, FO B cells interact with helper T cells to form GCs, produce high‐affinity antibodies to against foreign antigens, or differentiate into memory B cells to prevent the reinfection.^3^, ^53^ In the absence of CCR2, the reduction of FO B cells leads to a reduction in the proportion or number of plasma cells and plasmablasts under T‐cell‐dependent antigen stimulation. Biological signals tend to remain within a range to maintain immune homeostasis, abnormalities in either receptors or ligands can trigger certain thresholds leading to immune disorders. Overenhanced B‐cell signalling in CCR2‐deficient mice reduced antibody secretion during immune response.

In the process of B‐cell development and immune response, BCR signals activate numerous upstream and downstream signalling pathways in response to chemokines and receptors, thus achieving accurate and effective regulation. The regulation of CCR2 on B cells in the pathogenesis of SLE needs further study, finding targeted drugs from corresponding signalling pathways deserves to take a place in the treatment of autoimmune diseases.

## 5 CONCLUSIONS

CCR2 regulates BCR signalling to affect the biological activity of B cells, involving multiple signalling pathways dominated by MST1, mTORC1 and STAT1. It is worth investigating the effect of CCR2 in autoimmune diseases, so as to identify potential therapeutic targets.

## CONFLICT OF INTEREST

The authors declare no competing financial interests.

## Supporting information

FIGURE S1. CCR2 deficiency has no significant impact on B‐cell development in BM
FIGURE S2. CCR2 deficiency causes intrinsic peripheral B‐cell differentiation impairment
FIGURE S3. No lymphocyte infiltration was observed in liver, kidney and colon of mice
FIGURE S4. CCR2 deficiency produces B‐cell‐specific effects on peripheral differentiation
FIGURE S5. BCR proximal signalling is enhanced in *Ccr2*‐KO B cells
FIGURE S6. CCR2 deficiency enhances B‐cell metabolic signalling
FIGURE S7. *Ccr2*‐KO mice exhibit increased accumulation of F‐actin
FIGURE S8. CCR2 depletion triggers the activation of STAT1 to enhance BCR signalling

Supporting Information
