# Supplementary figures and images for: Involvement of MST1/mTORC1/STAT1 activity in the regulation of B‐cell receptor signalling by chemokine receptor 2
Source: Clin Transl Med. 2022 Jul 25;12(7):e887. doi: 10.1002/ctm2.887 (PMC9309749; doi:10.1002/ctm2.887)

Figure S1

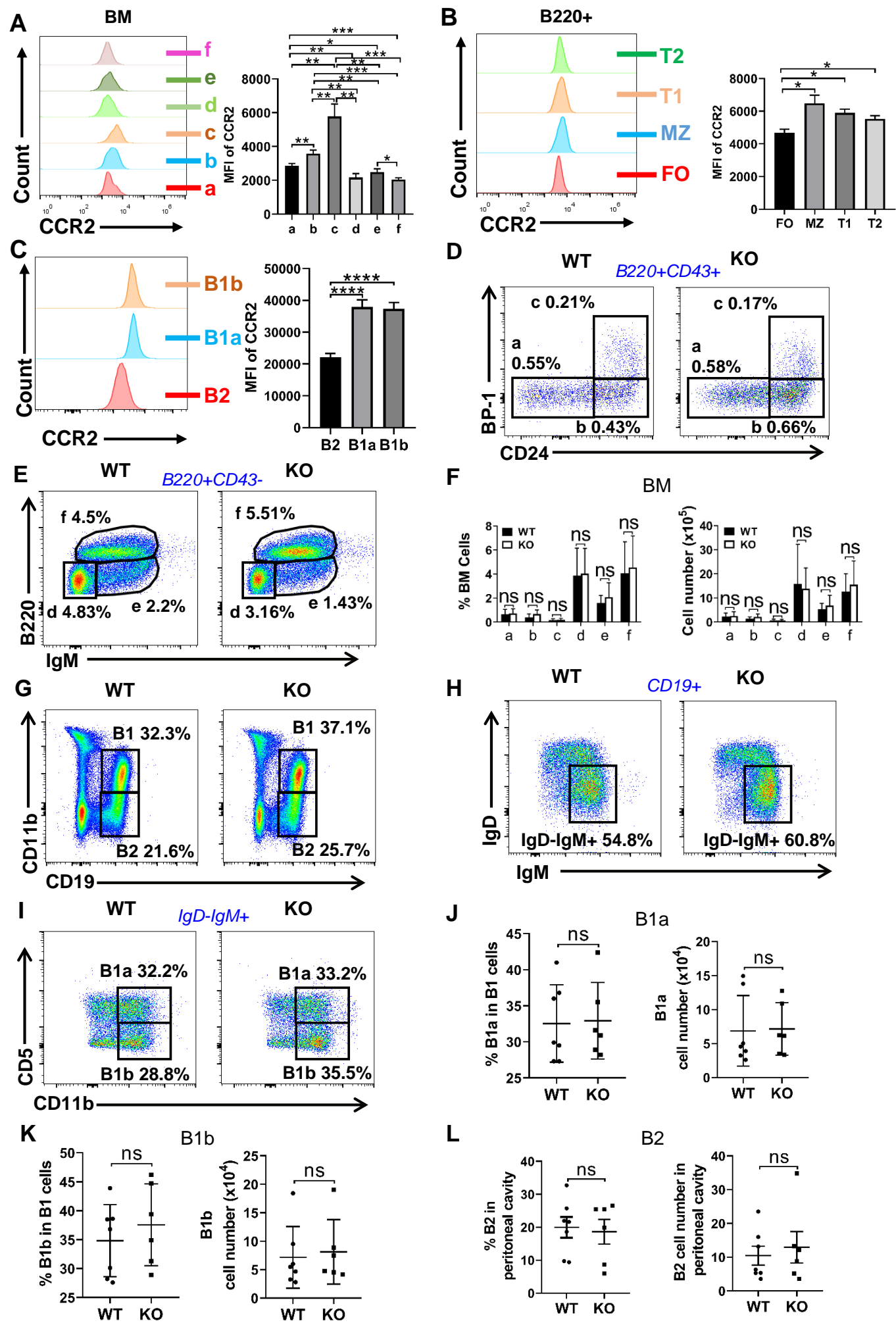

Figure S2

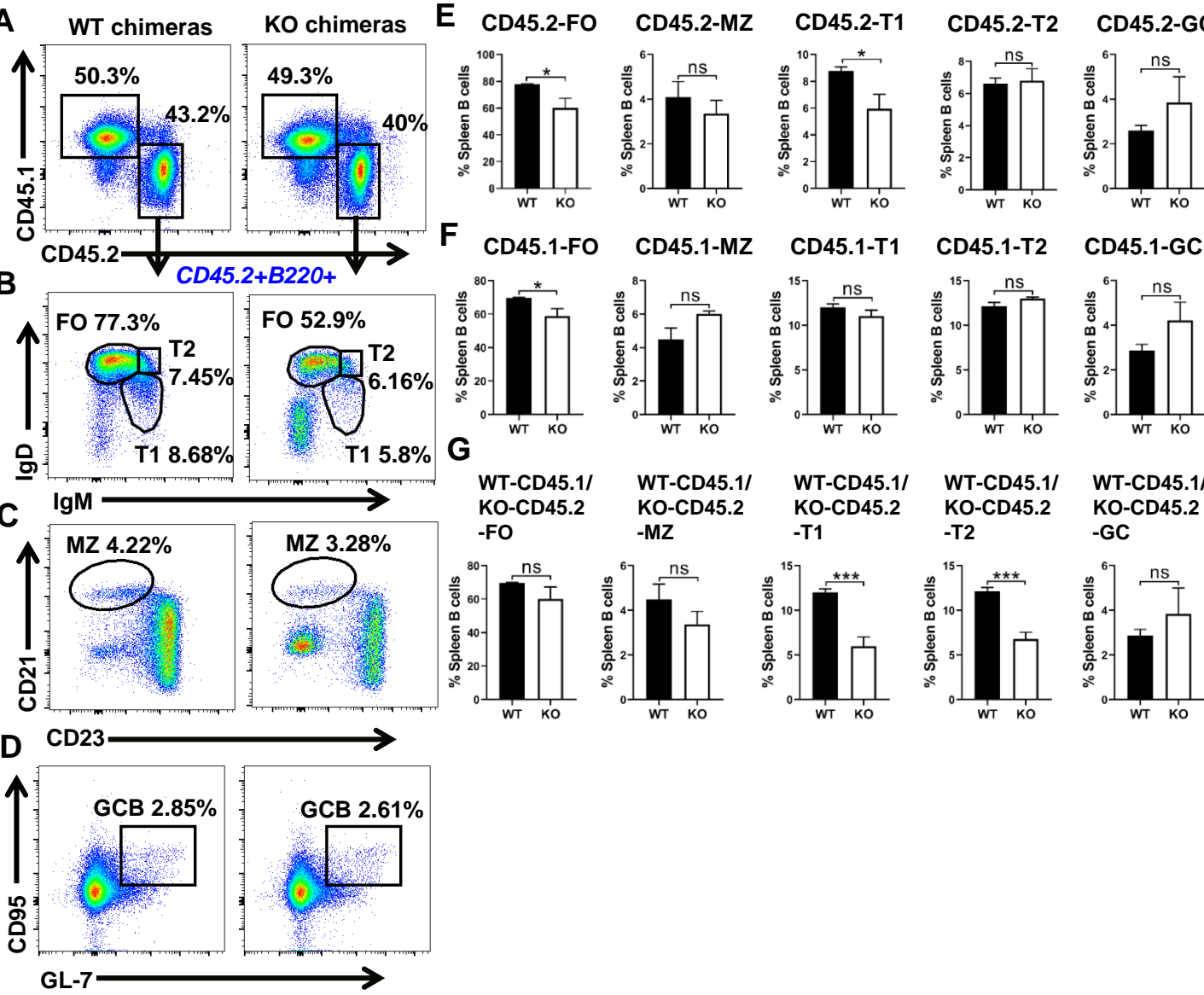

Figure S3

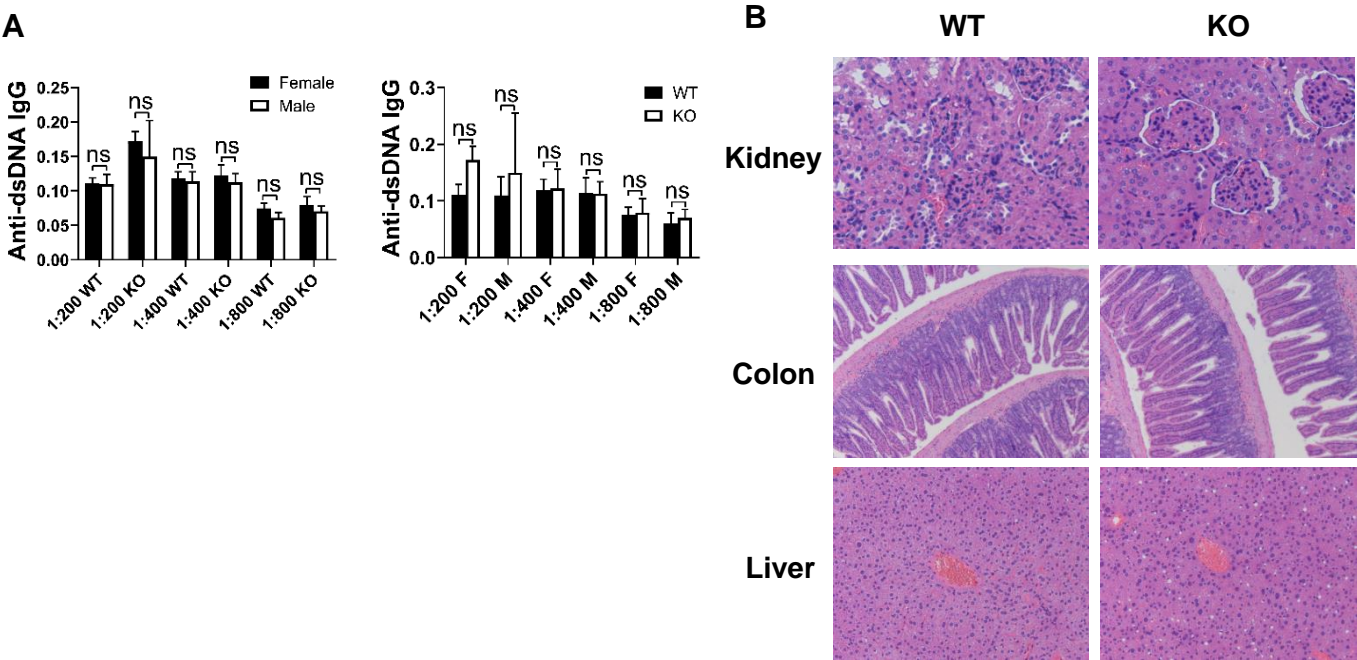

Figure S4

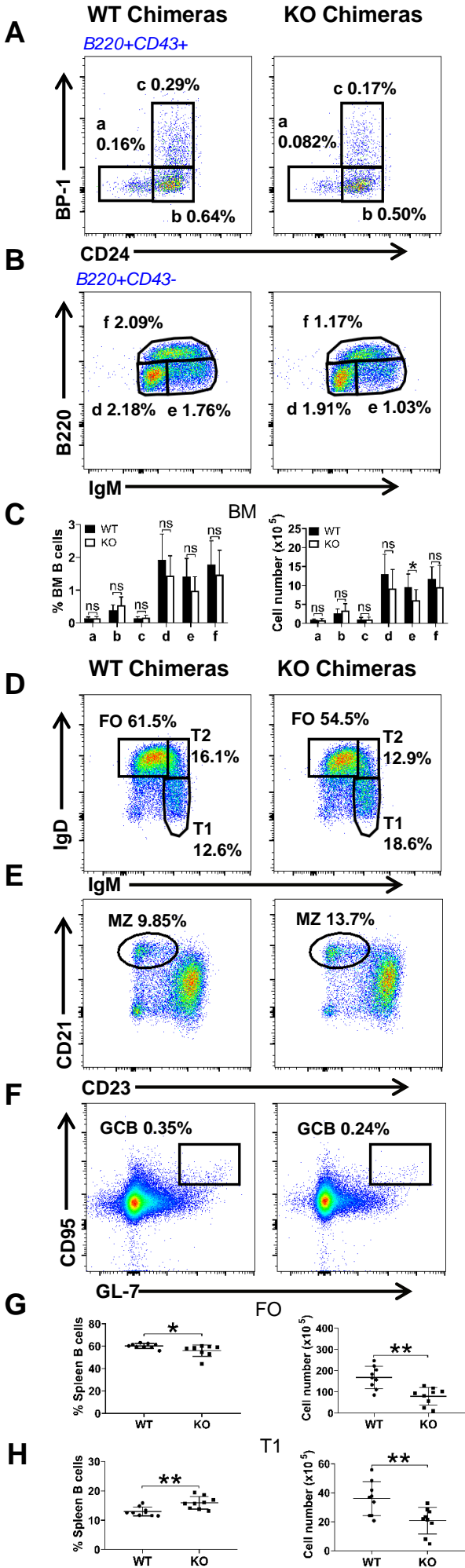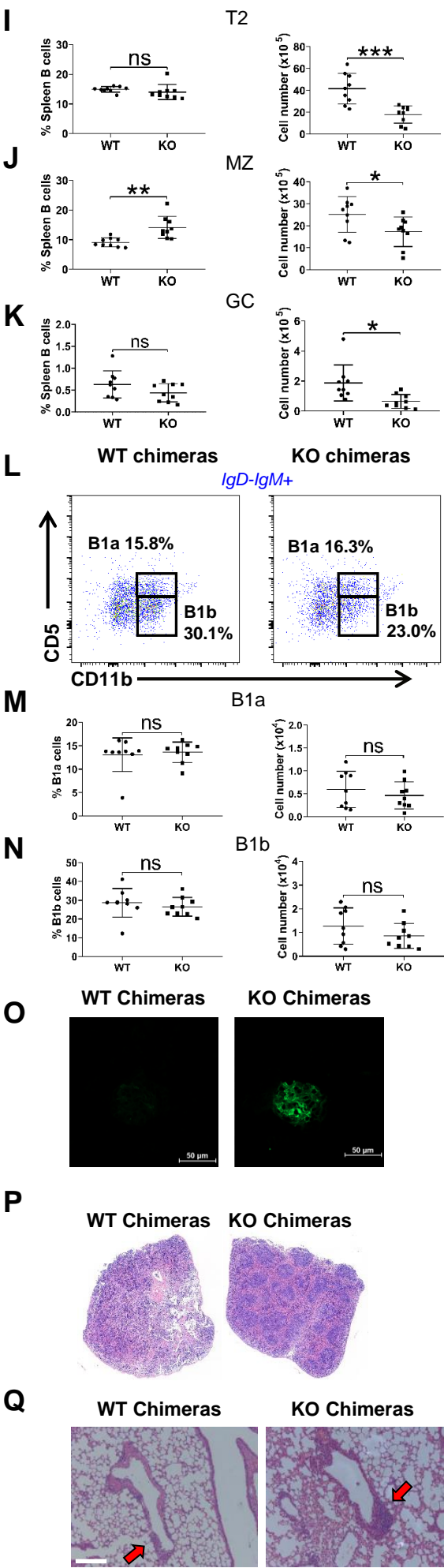

Figure S5

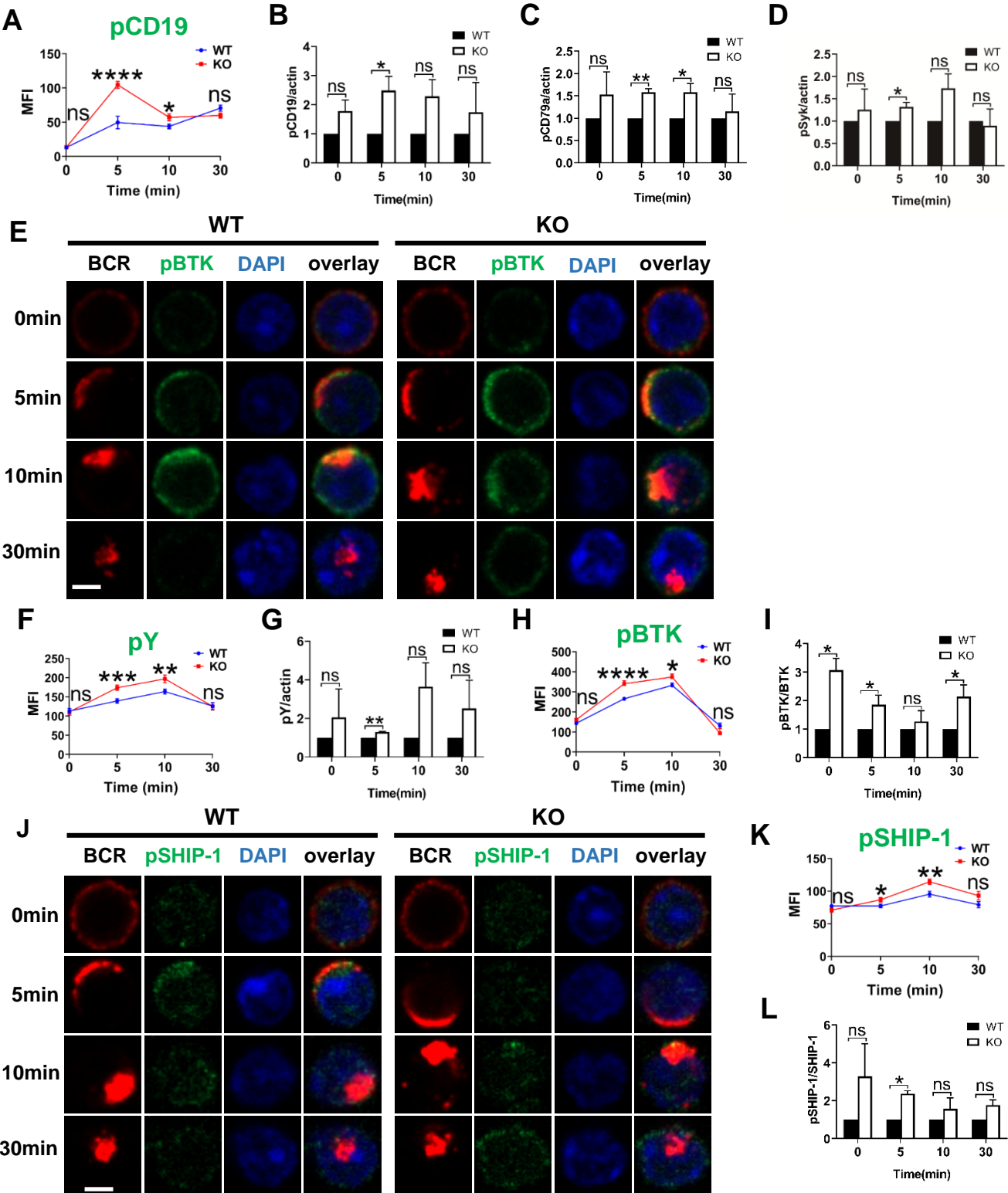

Figure S6

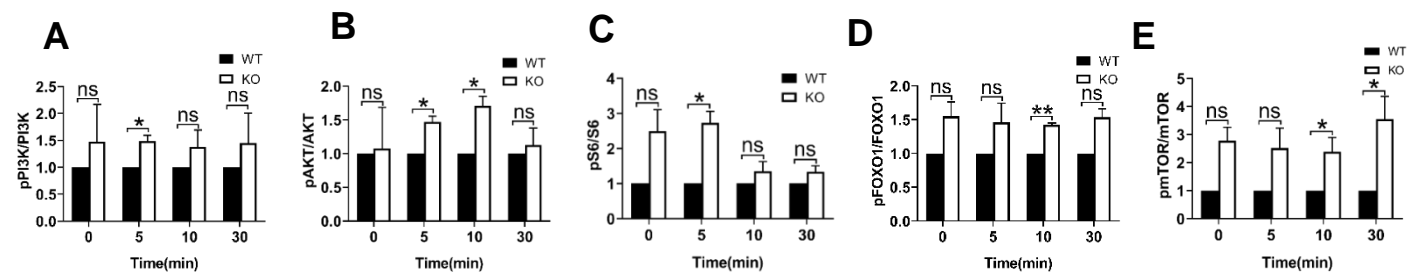

Figure S7

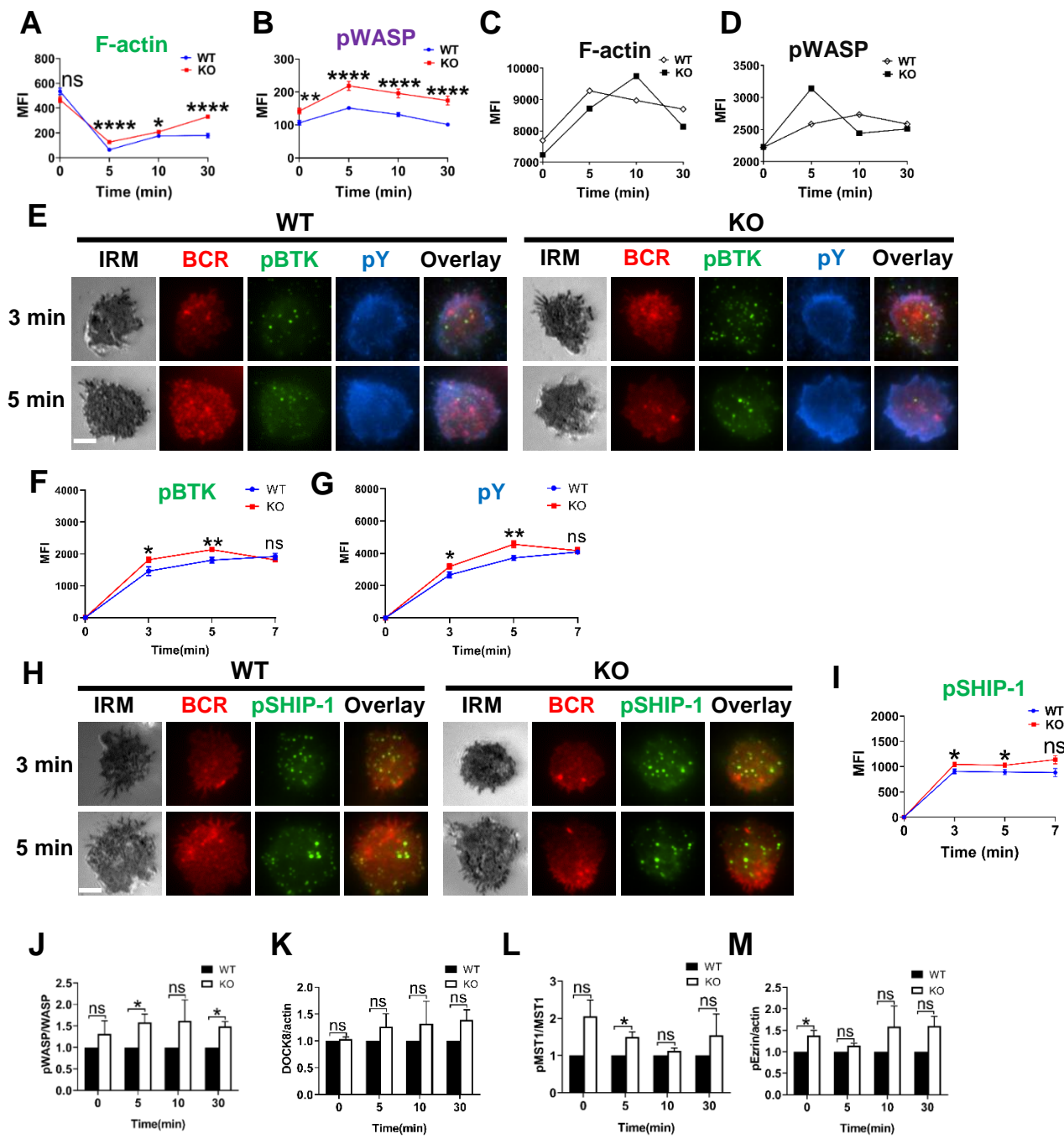

Figure S8

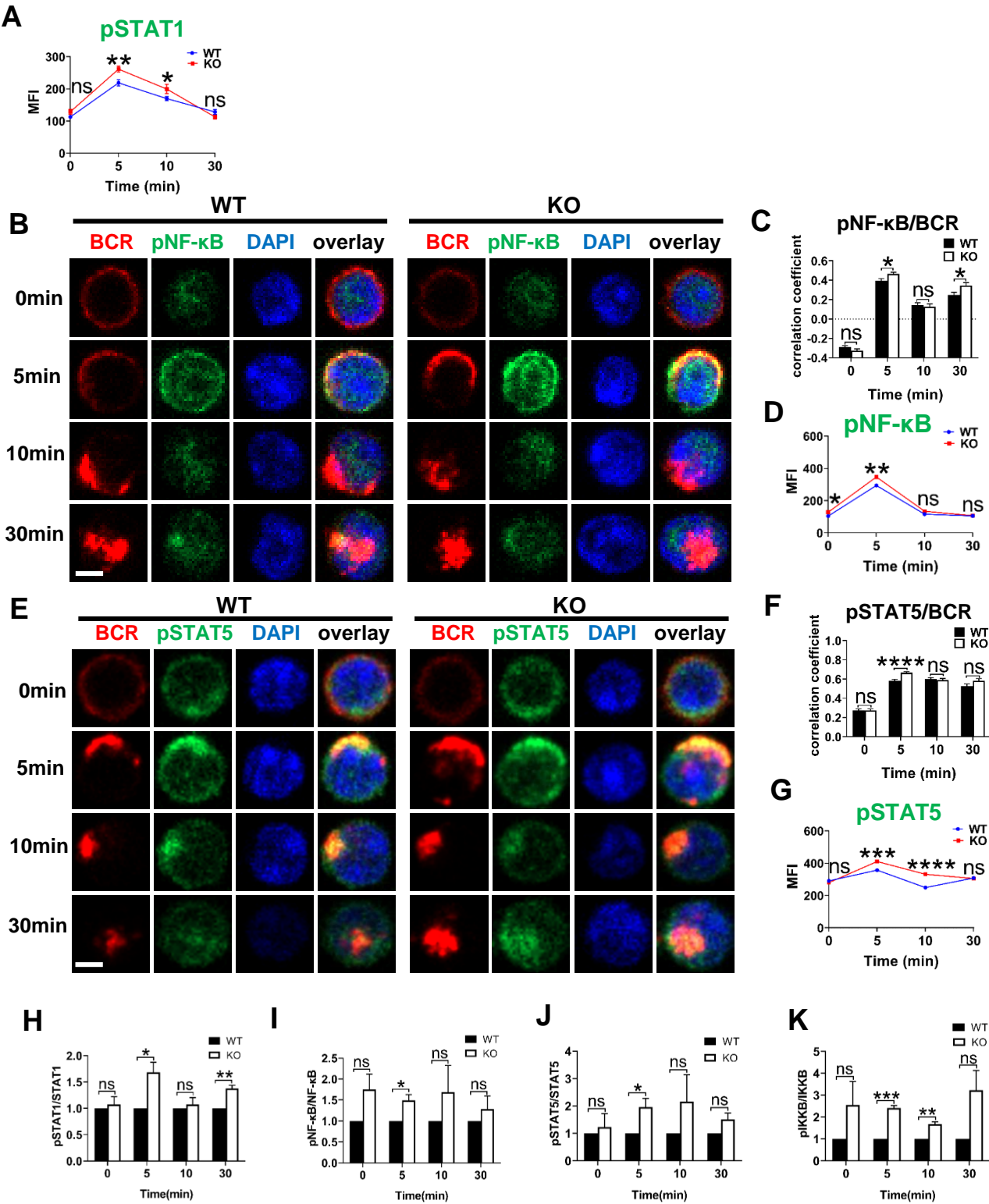

Supplement: Supplementary file 1 — FIGURE S1. CCR2 deficiency has no significant impact on B‐cell development in BM FIGURE S2. CCR2 deficiency causes intrinsic peripheral B‐cell differentiation impairment FIGURE S3. No lymphocyte infiltration was observed in liver, kidney and colon of mice FIGURE S4. CCR2 deficiency produces B‐cell‐specific effects on peripheral differentiation FIGURE S5. BCR proximal signalling is enhanced in Ccr2‐KO B cells FIGURE S6. CCR2 deficiency enhances B‐cell metabolic signalling FIGURE S7. Ccr2‐KO mice exhibit increased accumulation of F‐actin FIGURE S8. CCR2 depletion triggers the activation of STAT1 to enhance BCR signalling [file CTM2-12-e887-s001.pdf]
