# Supplementary material for: Involvement of MST1/mTORC1/STAT1 activity in the regulation of B‐cell receptor signalling by chemokine receptor 2
Source: Clin Transl Med. 2022 Jul 25;12(7):e887. doi: 10.1002/ctm2.887 (PMC9309749; doi:10.1002/ctm2.887)
Supplement: Supplementary file 2 — Supporting Information [file CTM2-12-e887-s002.docx]

**Figure legends of supplementary figures**

**Figure S1.** **CCR2 deficiency has no significant impact on B cell development in BM.**

(**A**) The expression and mean fluorescence intensity (MFI) of CCR2 on bone marrow (BM) B precursors of wildtype (WT) mice. (**B**) The expression and MFI of CCR2 on the splenic B subsets of WT mice. (**C**) The expression and MFI of CCR2 on the peritoneal cavity B subsets. (**D-E**) Representative dot plots of B precursors in BM. Shown in the box is the proportion of each B precursor in the total BM cells. Pre-pro B cell (a), pro B cell (b), early-pre B cell (c), late-pre B cell (d), immature B cell (e) and recirculating mature B cell (f). (**F**) Quantitative analysis of percentage and cell number of B precursors in BM (n = 8). (**G-I**) Representative dot plots of B2 (CD19^+^CD11b^-^), B1a (CD19^+^IgD^-^IgM^+^CD5^+^CD11b^+^) and B1b (CD19^+^IgD^-^IgM^+^CD5^-^CD11b^+^) cells in the peritoneal cavity. (**J-L**) Quantitative analysis of percentage and cell number of peritoneal cavity B1a, B1b and B2 cells (WT: n = 7, KO: n = 6). *P < 0.05, **P < 0.01, ***P < 0.001. ****P < 0.0001, ns: no statistical significance.

**Figure S2.** **CCR2 deficiency cause**s **intrinsic peripheral B cell differentiation impairment.**

(**A-D**) BM cells from WT or *Ccr2* KO (CD45.2) mice were mixed with that from WT (CD45.1) mice at a 50:50 ratio. Recipient WT mice (CD45.1) were irradiated with 7 Gy X-ray and injected intravenously with 5 × 10^6^ mixed cells. Representative dot plots of B cell subsets in CD45.2 WT and *Ccr2* KO chimeras. (**E**) Quantitative analysis of the proportion of FO B, MZ B, GC B, T1 and T2 cells in CD45.2 populations of WT mice (*n* = 5) and *Ccr2* KO (*n* = 3) mice. (**F**) Quantitative analysis of the proportion of FO B, MZ B, GC B, T1 and T2 cells in CD45.1 populations of WT mice (*n* = 5) and *Ccr2* KO (*n* = 3) mice. (**G**) Quantitative analysis of the proportion of FO B, MZ B, GC B, T1 and T2 cells in CD45.1 populations of WT mice (F) (*n* = 5) and CD45.2 populations of *Ccr2* KO mice (E) (*n* = 3). Error bars were shown as mean (± SD). *P < 0.05, ***P < 0.001, ns: no statistical significance.

**Figure S3. No lymphocyte infiltration was observed in liver, kidney and colon of mice.**

(**A**) Comparison of anti-dsDNA IgG in male and female mice (n = 9). (**B**) Kidney, colon, and liver of mice were harvested, sectioned, and stained with hematoxylin and eosin (HE) (10× objection). All images were representative images from three independent experiments, ns: no statistical significance.

**Figure S4. CCR2 deficiency produces B cell-specific effects on peripheral differentiation.**

BM cells from WT or *Ccr2* KO mice and μMT mice were mixed at a 20:80 ratio, then injected into irradiated recipient WT mice. Flow cytometry, immunohistochemistry and immunofluorescence were performed 8 weeks later. (**A-B**) Representative dot plots of B precursors in BM of WT and *Ccr2* KO chimeras. Shown in the box is the proportion of each B precursor in the total BM cells. Pre-pro B cell (a), pro B cell (b), early-pre B cell (c), late-pre B cell (d), immature B cell (e) and recirculating mature B cell (f). **(C)** Quantitative analysis of percentage and cell number of B precursors in BM (*n =* 9). **(D-F)** Representative dot plots of splenic B cell subsets of WT and *Ccr2* KO chimeras. Shown in the box is the proportion of each B cell subset in the total splenic B cells. **(G-K)** Quantitative analysis of percentage and cell number of splenic B cell subsets (*n =* 9). **(L)** Representative dot plots of B1a and B1b cells in the peritoneal cavity of WT and *Ccr2* KO chimeras. Shown in the box is the proportion of each B1 subset in the total B1 cells. **(M-N)** Quantitative analysis of percentage and cell number of B1a and B1b cells (*n =* 9). **(O)** IgG deposits in glomeruli of WT and *Ccr2* KO chimeras. (60× objective, scale bar = 50 μm) **(P)** Scan of spleen section of WT and KO chimeras after HE staining. **(Q)** HE staining of lung anatomical structure (10× objective, scale bar = 200 μm), the red arrows indicated lymphocytic infiltration around vessel. Error bars were shown as mean (± SD). Each symbol represents a mouse. *P < 0.05, **P < 0.01, ***P < 0.001, ns: no statistical significance.

**Figure** **S5. BCR proximal signaling is enhanced in *Ccr2* KO B cells.**

(**A**) Quantitative analysis of phosphorylated CD19 (pCD19) MFI. (**B**) Grayscale quantification of western blotting of pCD19. (**C**) Grayscale quantification of western blotting of pCD79a. (**D**) Grayscale quantification of western blotting of pSyk. (**E**) Representative CFm images of pBTK and BCR (60× objective, scale bar = 2.5 μm). (**F**) Quantitative analysis of pY MFI. (**G**) Grayscale quantification of western blotting of pY. (**H**) Quantitative analysis of pBTK MFI. (**I**) Grayscale quantification of western blotting of pBTK. (**J**) Representative CFm images of pSHIP-1 and BCR (60× objective, scale bar = 2.5 μm). (**K**) Quantitative analysis of pSHIP-1 MFI. (**L**) Grayscale quantification of western blotting of pSHIP-1. The number of cells analyzed for each parameter in CFm assay was 30-50. Error bars were shown as mean (± SD). *P < 0.05, **P < 0.01, ***P < 0.001, ****P < 0.0001, ns: no statistical significance.

**Figure** **S6. CCR2 deficiency enhances B cell metabolic signaling.**

(**A**) Grayscale quantification of western blotting of pPI3K. (**B**) Grayscale quantification of western blotting of pAKT. (**C**) Grayscale quantification of western blotting of pS6. (**D**) Grayscale quantification of western blotting of pFOXO1. (**E**) Grayscale quantification of western blotting of pmTOR. *P < 0.05, **P < 0.01, ns: no statistical significance.

**Figure** **S7. *Ccr2* KO mice exhibit increased accumulation of F-actin.**

(**A**) The MFI of F-actin. (**B**) The MFI of pWASP. (**C-D**) Phosphoflow cytometry detection of pWASP and F-actin levels in B220^+^ B cells. (**E**) Representative TIRFm images of pY and pBTK at 3 and 5 min of activation (100× objective, scale bar = 2.5 μm). **(F)** The MFI of pBTK. **(G)** The MFI of pY. **(H)** Representative TIRFm images of pSHIP-1 at 3 and 5 min of activation (100× objective, scale bar = 2.5 μm). **(I)** The MFI of pSHIP-1. **(J)** Grayscale quantification of western blotting of pWASP. (**K**) Grayscale quantification of western blotting of DOCK8. (**L**) Grayscale quantification of western blotting of pMST1. (**M**) Grayscale quantification of western blotting of pEZRIN. The number of cells analyzed for each parameter in TIRFm assay was 30-50. Error bars were shown as mean (± SD). *P < 0.05, **P < 0.01, ****P < 0.0001, ns: no statistical significance.

**Figure** **S8. CCR2 depletion triggers the activation of STAT1 to enhance BCR signaling**

(**A**) The MFI of pSTAT1. (**B**) Representative CFm images of pNF-κB (60× objective, scale bar = 2.5 μm). **(C)** Quantitative analysis of the colocalization between pNF-κB and BCR. **(D)** The MFI of pNF-κB. **(E)** Representative CFm images of pSTAT5 (60× objective, scale bar = 2.5 μm). **(F)** Quantitative analysis of the colocalization between pSTAT5 and BCR. (**G**) The MFI of pSTAT5. (**H**) Grayscale quantification of western blotting of pSTAT1. (**I**) Grayscale quantification of western blotting of pNF-κB. (**J**) Grayscale quantification of western blotting of pSTAT5. (**K**) Grayscale quantification of western blotting of pIKKB. The number of cells analyzed for each parameter in CFm assay was 30-50. Error bars were shown as mean (± SD). *P < 0.05, **P < 0.01, ***P < 0.001, ****P < 0.0001, ns: no statistical significance.
